# Systematic Review and Meta-Analysis of Lipid Emulsion for Non-Local Anesthetic Poisoning

**DOI:** 10.1007/s13181-026-01151-7

**Published:** 2026-08-27

**Authors:** Zhengxin Tang, Megan Zhao, Alexandra Barabanova, Quincy McCrary, Michael R. Fettiplace

**Affiliations:** 1 Department of Anesthesiology, University of Illinois at Chicago, IL Chicago, USA; 2 Library of the Health Sciences, University of Illinois at Chicago, Chicago, IL USA

**Keywords:** Complication, Toxicity, Lipid emulsion, Organophosphate, Aluminum phosphide, Mortality, Antipsychotic, Intubation

## Abstract

**Introduction:**

Practitioners routinely use intravenous lipid emulsion for local anesthetic systemic toxicity, but its role in non-local anesthetic poisonings remains unclear. We conducted a systematic review and meta-analysis comparing lipid emulsion with standard care for non-local anesthetic toxicities.

**Methods:**

We searched PubMed, Embase, Scopus and Web of Science from January 1st 2010 until March 16th 2026 for human randomized controlled trials evaluating lipid emulsion for non-local anesthetic poisoning. Volunteer studies and pediatric-only studies were excluded. The primary outcome was intensive care unit admission (with intubation as a surrogate for unit-level needs). Secondary outcomes included mortality, seizures, and hospital length of admission. Risk of bias was assessed using the Cochrane Risk of Bias 2 tool. Random-effect meta-analysis was conducted. Heterogeneity was analyzed using Cochran’s Q and I^2^.

**Results:**

Twelve studies with 816 patients were included, predominantly using 20% SMOFlipid® 280 – 500 mL. Lipid emulsion reduced intensive care unit admission compared to standard care (RR=0.70; 95% CI: [0.53, 0.93], 8 studies, n=369) with moderate heterogeneity (Q=9.43, p=0.02; I^2^=32.12%). Lipid emulsion also reduced mortality (RR=0.65; 95% CI: [0.53, 0.80]) and length of admission (Mean diff. =-34.16; 95% CI: [-42.29,-26.02]). Seizure incidence was lowered but not significantly (RR=0.27; 95% CI [0.06, 1.26]).

**Discussion:**

Intravenous lipid emulsion is associated with reduced intensive care unit admission and mortality for aluminum phosphide and organophosphate poisonings and a reduced length of admission for aluminum phosphide, organophosphate, and antipsychotic poisonings. The evidence is limited by study heterogeneity and study quality. Larger, high-quality trials are needed to confirm benefit.

**Registration Number:**

CRD42025645519

**Supplementary Information:**

The online version contains supplementary material available at https://doi.org/10.1007/s13181-026-01151-7.

## Introduction

Practitioners utilize intravenous lipid emulsion (ILE) as a first-line therapy for local anesthetic systemic toxicity (LAST) [1], with efficacy supported by both preclinical studies [2] and case reports [3, 4]. Beyond LAST, medical toxicologists, emergency physicians, and intensivists use ILE for acute poisonings. Lipid emulsion produces benefits via a multimodal mechanism [5, 6] including both a redistribution effect referred to as the “lipid shuttle” [7] and a direct cardiovascular benefit [8]. These two mechanisms also occur in non-local anesthetic toxicity, with redistributive effects observed in animal models of paliperidone and amiodarone toxicity [9, 10] along with blood pressure benefits seen in retrospective studies of poisoned human patients [11]. Following the introduction of ILE as a treatment for LAST, mortality and reporting of LAST from long-acting local anesthetics dropped precipitously [12, 13]. However, less data exists to support the use of ILE in non-local anesthetic poisonings. Meta-analyses of animal studies demonstrate the benefit of ILE in non-local anesthetic overdose [14], with frequent case reports supporting its clinical efficacy [15] but in 2016, a professional workgroup of toxicologists recommended against the use of ILE except in extreme circumstances [16].

Since that recommendation, several groups evaluated the efficacy of ILE via randomized controlled trials for the non-local anesthetic toxicities, including organophosphate [17–20], antipsychotic [21, 22], tramadol [23, 24] and aluminum phosphide [25–27]. Two recent publications synthesized the emerging clinical evidence on ILE use in acute poisoning: Bilvanisi et al. [28] published a narrative review summarizing case-based data supporting ILE but did not perform a formal quantitative analysis or structured risk-of-bias assessment. Similarly, Koh et al. [29] conducted a systematic review of randomized controlled trials of ILE for antipsychotic and sedative poisonings and identified five eligible studies; however, due to non-uniform reporting of continuous outcomes and marked heterogeneity in endpoints, the authors did not perform a pooled meta-analysis, instead presenting study-level findings descriptively. Additionally, their review focused on changes in Glasgow Coma Scale (GCS) as the main outcome. Given the limitations of prior work, we conducted a systematic review and meta-analysis of ILE in non-local anesthetic overdose. Since many of these overdoses occur in resource-constrained settings, we chose intensive care unit (ICU) admission as our primary outcome, with intubation as a surrogate variable for unit-level needs. Finally, we included all toxic overdoses since the redistributive and cardiotonic effects should be less specific to the type of intoxication.

## Methods

### Study Design

This systematic review and meta-analysis were conducted in accordance with the Preferred Reporting Items for Systematic Reviews and Meta-Analyses (PRISMA) guidelines. Randomized trials comparing the efficacy of ILE to placebo or standard-of-care treatment for xenobiotic toxicity were included. The full protocol is available in the International Prospective Register of Systematic Reviews under PROSPERO number CRD42025645519.

### Study Screening

An information specialist searched four electronic databases (PubMed, Embase, Scopus and Web of Science) on October 21 st, 2024, filtered from 2010 to 2024 using a combination of medical subject headings and free text terms to identify publications about ILE and toxicity, including “systemic toxicity”, “toxicity”, “intoxication”, “poisoning”, “Intralipid”, “lipid emulsion”, “smoflipid”, “clinoleic”, “nutralipid”, “omegaven”; no language restrictions were applied. A second search using the same queries was conducted on March 16th, 2026 to capture publications from 2024 to 2026. We identified additional sources through expert recommendation and hand searching. The complete search strategy for each database is provided in the supplemental material. Following removal of duplicate records, two reviewers (ZT, MZ) screened titles and abstracts followed by full-text reviews in Covidence (Melbourne, Australia). Discrepancies in judgment were resolved by a third reviewer (MRF). Non-English articles were manually translated using Google Translate.

Eligible studies were peer-reviewed randomized controlled trials published between 2010 and 2026 that included the use of any ILE therapy for the treatment of xenobiotic overdose. Exclusion criteria were reviews, observational studies, meta-analyses, commentaries, opinion pieces, abstracts, case reports, animal studies, studies focused solely on LAST, and pediatric-only populations. Studies that included both adults and children were eligible for inclusion. Separate syntheses were conducted for each predefined outcome, including ICU admission and/or intubation, mortality, seizures, and length of admission. Only studies reporting data for a given outcome were included in its analysis. Studies that assessed the outcome but reported no events or identical values across groups were recorded but excluded from the analysis for that outcome.

Following screening, data was extracted by two authors (ZT, MZ) with mediation by a third author (MRF). Extracted data included study settings, primary author contact, type of xenobiotic toxicity, lipid dosing, population, baseline population characteristics, need for ICU admission, need for invasive ventilation, mortality, seizures, length of admission, GCS, need for vasopressor support, mean arterial pressure, systolic blood pressure, diastolic blood pressure, heart rate, triglycerides, aspartate aminotransferase and alanine aminotransferase. Continuous outcomes were recorded as means and standard deviations, and dichotomous outcomes as event counts.

### Outcome and Analysis

The primary outcome was a need for ICU admission, with invasive ventilation as an additional surrogate for ICU level care. Secondary outcomes, if available, included mortality, seizures, length of admission, GCS, need for vasopressor support, mean arterial pressure, systolic blood pressure, diastolic blood pressure, heart rate, triglycerides, aspartate aminotransferase and alanine aminotransferase. All statistical analyses and figures were generated using SPSS. For length of stay, Wan et al.’s method [30] for estimating mean and standard deviation was used. For binary outcomes, results were reported as relative risk, and effect size. For continuous outcomes (e.g. length of admission), effect sizes were calculated as standardized mean differences using a random-effects Empirical Bayes model. Heterogeneity was calculated using I^2^. A random-effects empirical Bayes model was used, given anticipated between-study heterogeneity; subgroup analysis based on type of intoxicant was also performed (grouped into organophosphate, antipsychotic, aluminum phosphide, and mixed-intoxication). Publication bias was evaluated using funnel plots, Egger’s test, Begg’s test and Galbraith plots for all applicable analyses. L’Abbé plots were used to visualize consistency and direction of treatment effects across studies. Risk of bias was evaluated using Cochrane Risk of Bias 2 (RoB2) tool. An exploratory assessment was conducted using the Grading of Recommendations Assessment, Development and Evaluation (GRADE) framework, with overall certainty of evidence categorized as high, moderate, low, or very low. We conducted an exploratory leave-one-out sensitivity analysis for all positive outcomes and a sensitivity analysis for length-of-stay, excluding studies that required Wan et al.’s method.

## Results

**Fig. 1:**
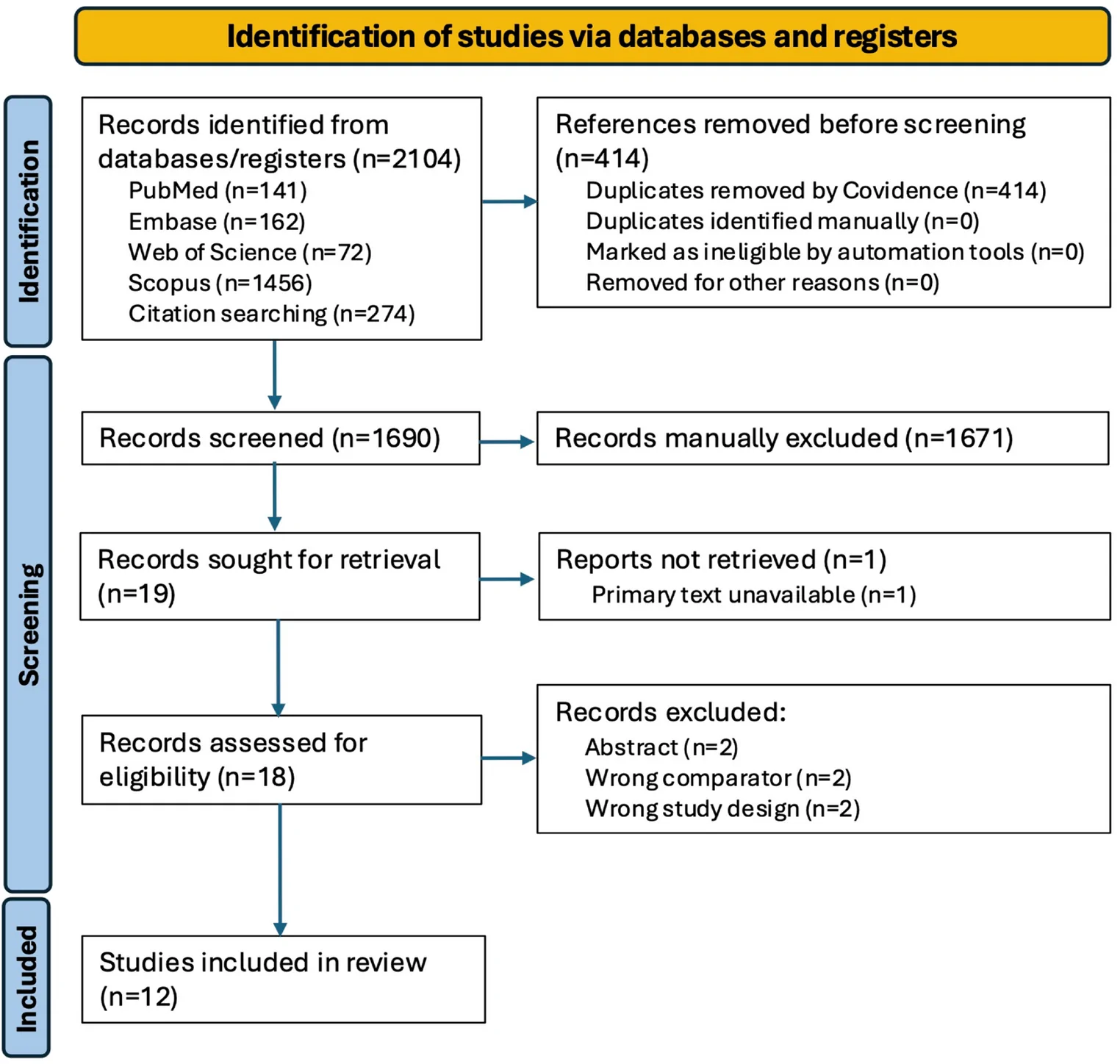
PRISMA flow chart of study screening process

A total of 2104 records were identified through database searching. Study screening progress was documented in a PRISMA flow chart (Fig. 1). A total of 12 studies with 816 patients met criteria for inclusion [18–27, 31, 32] in quantitative meta-analysis (Table 1 & Supplemental Tables 1 & 2). Data from Gheat et al. [25] were analyzed as two independent comparisons, as the study reported separate outcomes for moderate and severe aluminum phosphide poisoning. Certain secondary outcomes (aspartate aminotransferase, alanine aminotransferase, need for vasopressor support) were not evaluated given lack of reporting in included studies. The primary formulation was SMOFlipid^®^ 20% with consistent bolus dosing of 1.5mL/kg or 100mL and a total dose of ~ 280–500mL. Only three studies used volume control for the lipid. Regarding additional treatment, most studies (10 out of 12) used gastric decontamination/lavage. All organophosphate studies used atropine, and all aluminum phosphide cases used vasopressors/inotropes and sodium bicarbonate (Supplemental Table 2).

**Table 1:** Study characteristics [Control/ILE]

Study	Lipid	Bolus	Infusion/Duration	Total dose^#^	Patients	Age^¥^	ICU admit*	Mortality	Seizures	Admit duration (hours)¥
*Tramadol*					*100/100 tramadol total patients*					
Behnoush 2024	20% SMOFlipid^®^	100mL	250 mL/15 min	350	60/60	28 ± 4/ 23 ± 7	/	/	30/16	/
Kazemifar 2021	20% Intralipid^®^	3.6 mL/kg	8.4 mL/kg/3 h	840	40/40	23 ± 6/ 25 ± 8	/	/	15/0	/
*Organophosphates*					*148/146 organophosphate total patients*					
Pannu 2022	20% ILE	100mL	100 mL/6 h	200	22/23	27 (20–33)/ 23 (20–43)	10/12	3/1	1/0	/
Majeed 2019	20% ILE	1.5 mL/kg	1.5 mL/kg/hr/5 h	210	50/50	28 ± 10/ 27 ± 7	50/42	20/9	/	140 ± 72/117 ± 41
Guo 2015	20% Fat emulsion		250mL/5 h	250	59/56	35 ± 9/ 35 ± 9	/	/	/	209 ± 60/175 ± 50
Minjun 2013	30% Fat emulsion		250mL/3 h	375	17/17	40 ± 17/ 41 ± 15	/	8/3	/	241 ± 99/219 ± 85
*Antipsychotics*					*40/40 Antipsychotic total patients*					
Elgazzar 2021 §	20% SMOFlipid^®^	1.5 mL/kg	6 mL/kg/1 h	525	20/20	18 (5–22)/ 17 (7–22)	4/1	/	/	70 ± 28/29 ± 18
Basiouny 2022	20% SMOFlipid^®^	1.5 mL/kg	6 mL/kg/1 h	525	20/20	14 (5–22)/ 10 (4–19)	3/1	/	/	57 ± 40/33 ± 23
*Aluminum phosphide*					*107/105 Aluminum phosphide total patients*					
Gheat 2024 (moderate)	20% SMOFlipid^®^	1.5 mL/kg	0.25 mL/kg/min/10 min^&^	280	16/16	21 (18–33)/ 20 (16–29)	5/4	12/9	/	/
Gheat 2024 (severe)	20% SMOFlipid^®^	1.5 mL/kg	0.25 mL/kg/min/for 10 min^&^	280	16/16	23 (18–33)/ 22 (18–38)	8/4	15/11	/	/
ELabdeen 2020	20% SMOFlipid^®^		10 mL/hr / 10–100 h (median 30 h)	300	25/25	Range <20–50/Range <20–50	23/9	20/9	/	104 ± 23/63 ± 13
Bhatia 2026	20% Soybean oil emulsion	250mL	250 mL/30 min	500	50/48	35(Range 25–44)/ 32(Range 25–39)	/	31/11	/	/
*Mixed*					*15/15 Mixed toxicity total patients*					
Taftachi 2012	10% Intralipid^®^		10 mL/kg	350	15/15	28 ± 11/ 23 ± 5	9/6	/	/	/
OVERALL	Median dose [IQR] : 350 [280–500] mL				410/406 Total patients (816 total)					

# - Normalized to a for 70 kg patient receiving 20% over 24 hours

&: Can re-bolus or double the infusion rate

ILE: Intravenous lipid emulsion (not otherwise specified)

§-includes only clozapine,

*: ICU admission or intubation as a surrogate

^+^: As calculated by Wan et al.;

¥: reported as mean +/- STD or median (IQR)

### Risk of Bias

Evaluation with the RoB2 tool demonstrated an overall unclear to high risk of bias (Supplemental Fig. 1), mainly due to absent prespecified protocols, possible selective outcome reporting, and inadequate randomization. Most trials did not use an active placebo, and only Behnoush et al. [24] was double-blinded, raising the risk for detection bias (Supplemental Table 2).

### Primary Outcome: ICU Admissions and/or Need for Intubation

**Fig. 2:**
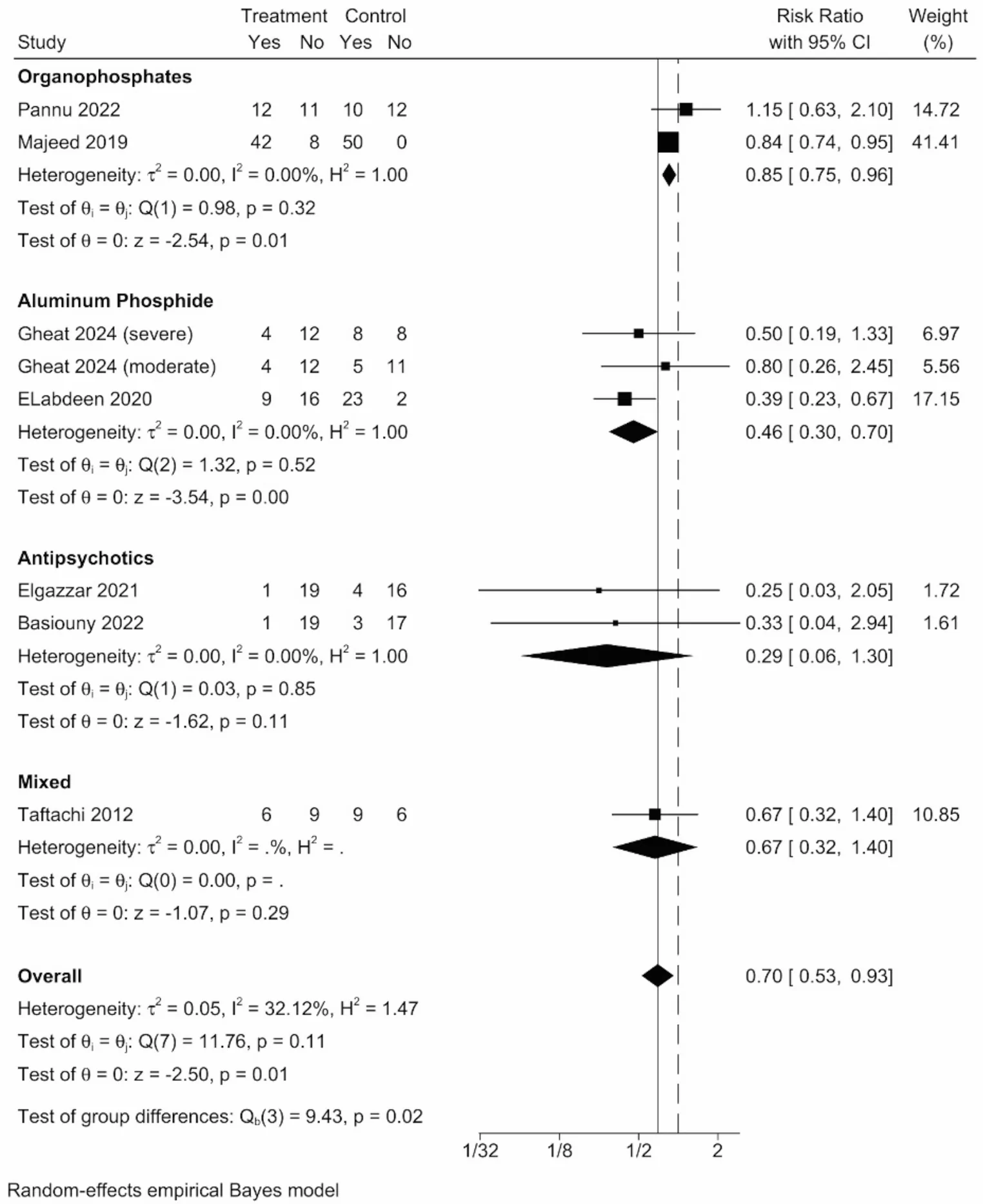
Forest plot of ICU admission rates comparing adjuvant intravenous lipid emulsion (treatment) with standard resuscitation (control)

Eight studies reported ICU admission or intubation (Fig. 2, *n* = 369 patients). Overall, ILE demonstrated a reduction in invasive ventilation compared to control (RR = 0.70; 95% CI: [0.53, 0.93]), with moderate heterogeneity across studies (I^2^ = 32.12%). Subgroup differences indicated the treatment effect was moderated by drug class (Q_b_(3) = 9.43, *p* = 0.02). The most pronounced benefit was seen in the aluminum phosphide subgroup (RR = 0.46; 95% CI: [0.30, 0.70]), followed by the organophosphate subgroup (RR = 0.85; 95% CI: [0.75, 0.96]). The antipsychotic subgroup showed a large but non-significant reduction in invasive ventilation (RR = 0.29; 95% CI: [0.06, 1.30]), single mixed-poisoning study did not reach significance (RR = 0.67; 95% CI: [0.32, 1.40]). Visual inspection of the funnel plot (Supplemental Fig. 2) shows mild asymmetry, with smaller studies generally reporting larger treatment effects. The Galbraith plot shows all studies within the 95% confidence interval and a negatively sloped regression line, with one study above the no-effect line (Supplemental Fig. 3). There was no evidence of small-study effects based on Begg’s test (z = − 1.11; *p* = 0.39) or Egger’s regression test (β₁=−0.82; SE = 0.72; z = − 1.15; *p* = 0.25). The L’Abbé (Supplemental Fig. 4) demonstrated consistent effects without obvious heterogeneity. Leave one out analysis for ICU admission did not impact the overall odds ratio but the exclusion of Majeed et al. [19] made organophosphate non-significant (Supplemental Fig. 5) and the exclusion of Elabdeen et al. [26] made aluminum phosphide non-significant (Supplemental Fig. 6).

### Secondary Outcomes: Mortality

**Fig. 3:**
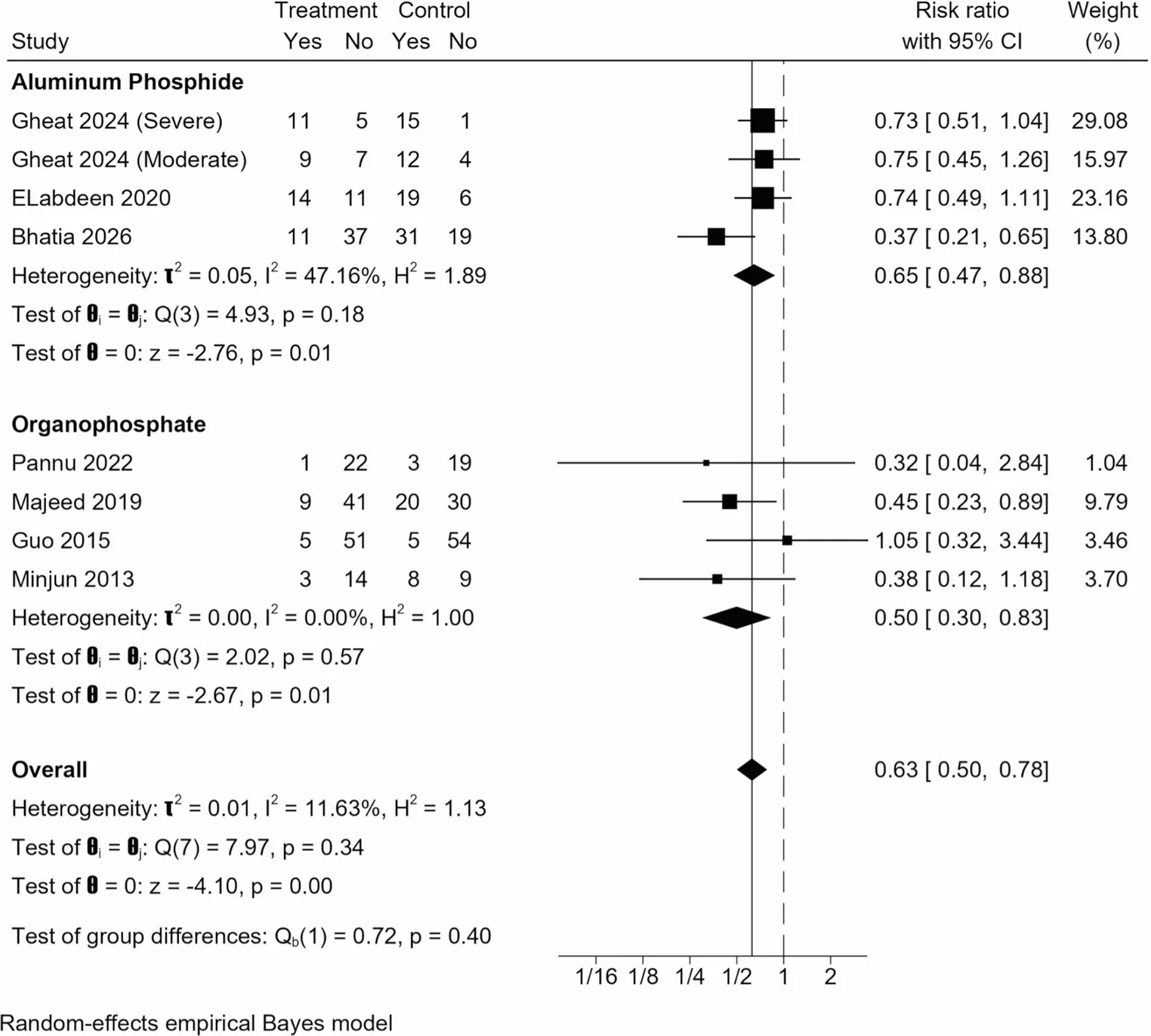
Forest plot of mortality rates comparing adjuvant intravenous lipid emulsion (treatment) with standard resuscitation (control)

Eight studies reported mortality data (Fig. 3, *n* = 506 patients). Only aluminum phosphide and organophosphate studies reported data for this outcome. ILE treatment was associated with reduced mortality rates compared to control (RR = 0.63; 95% CI: [0.50, 0.78]) with negligible heterogeneity (I^2^ = 11.63%). Similar effects were seen in the organophosphate subgroup (RR = 0.50; 95% CI: [0.30, 0.83]) and aluminum phosphide subgroup (RR = 0.65; 95% CI: [0.47, 0.87]). Visual inspection of funnel plot shows minimal asymmetry (Supplemental Fig. 7). Neither Egger’s test (β=-0.87, *p* = 0.2957) nor Begg’s test (*p* = 0.5362) demonstrated evidence of small-study effects. The Galbraith plot (Supplemental Fig. 8) showed all studies within the 95% CI with no extreme outliers. L’Abbé plot (Supplemental Fig. 9) echoes this pattern, with a consistent trend toward reduced mortality with ILE treatment. Leave one out analysis of mortality did not impact the overall odds ratio, but the exclusion of Majeed [19] made organophosphate non-significant (Supplemental Fig. 10).

### Secondary Outcome: Length of Admission

**Fig. 4:**
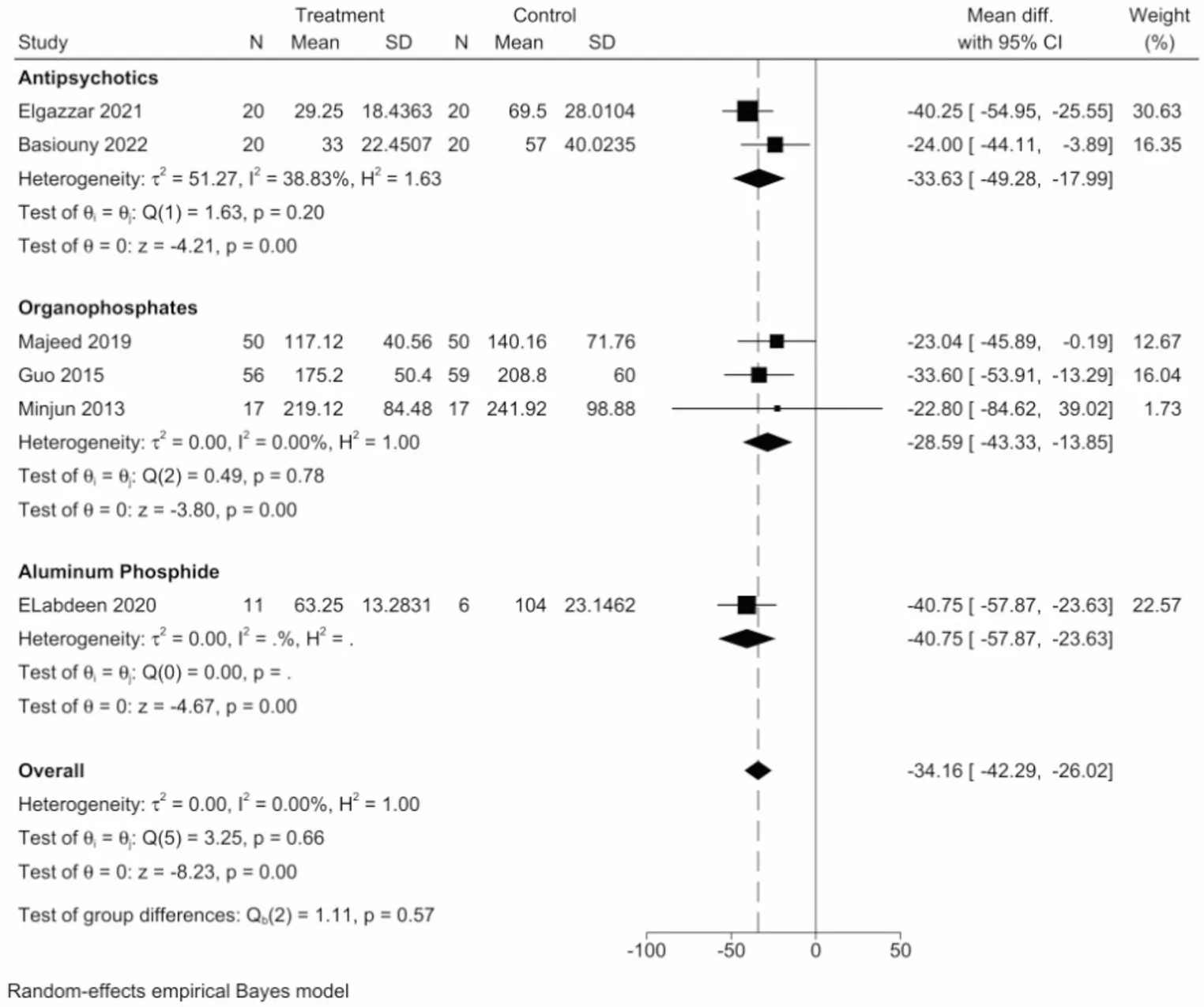
Forest plot of length of admission comparing adjuvant intravenous lipid emulsion (treatment) with standard resuscitation (control)

Including data generated using Wan et al.’s method [30], six studies reported data on length of admission (Fig. 4, *n* = 379 patients). Pooled analysis demonstrated a significant reduction in length of admission with ILE treatment (Mean diff. =-34.16 h; 95% CI: [-42.29, -26.02]), with no heterogeneity (I^2^ = 0%). All subgroups demonstrated reductions in length of admission, including antipsychotics (Mean diff. = -33.63 h, 95% CI: [-49.28, -17.99]), organophosphates (Mean diff. = -28.59, 95% CI: [-43.33, -13.85]), and aluminum phosphides (Mean diff. = -34.16 h, 95% CI: [-42.29, -26.02]). Egger’s regression test had some evidence of small-study effects (β = -3.70; *p* = 0.0015), while Begg’s test did not show significant small-study effects (*p* = 0.1078). Funnel plot analysis (Supplemental Fig. 11) demonstrated no asymmetry. The Galbraith plot (Supplemental Fig. 12) showed all studies falling within the 95% CI band, with effects consistently favoring ILE across all included studies. Leave-one-out analysis of length of stay and exclusion of interpolated data (Supplemental Fig. 13) did not impact the overall odds ratio.

### Secondary Outcomes: Seizures

Three studies reported seizure incidence (Supplemental Fig. 14, *n* = 245), with Behnoush et al. [24] contributing the most weight (61.62%). ILE treatment was associated with fewer seizures compared with control but did not reach significance (RR = 0.27; 95% CI [0.06, 1.26]); moderate heterogeneity was present (I^2^ = 44.3%). No seizure events were reported in the ILE group in either Pannu et al. [18] or Kazemifar et al. [23]. Given the low number of included studies, Funnel plot (Supplemental Fig. 15), Galbraith plot (Supplemental Fig. 16), and L’Abbé plot (Supplemental Fig. 17) could not be reliably interpreted for publication bias.

### Other Outcomes

Several secondary outcomes had an insufficient number of studies or non-aligned sampling points to conduct a meta-analysis. Variables include GCS, triglycerides, systolic blood pressure, diastolic blood pressure and mean arterial pressure (Supplemental Table 1). Although there was not enough data for a formal meta-analysis, comparisons between Behnoush et al. [24], Elgazzar et al. [21], and Basiouny et al. [22] consistently showed a faster improvement in GCS scores in the ILE group compared with the control group. In Behnoush et al. [24], mean GCS increased by 2 points in the ILE group (10 to 12) compared to a 0.5-point increase in the control group (9.5 to 10) at the same timepoint (12 h after baseline). Similarly, Elgazzar et al. [21] reported a larger improvement in the ILE group (8.8 to 14.3) compared with the control group (8.5 to 9.6). While there was less of a difference in Basiouny et al. [22], follow-up GCS values were reported as medians rather than means, limiting direct comparison. Other continuous outcomes, such as serum triglycerides and blood pressure measurements, did not show meaningful changes with ILE therapy.

### GRADE Assessment

An exploratory GRADE assessment was performed across four outcomes: need for ICU admission, mortality, length of hospital admission, and seizures (Supplemental Fig. 18). For ICU admission, ILE was associated with a reduction in risk corresponding to 183 fewer events per 1,000 patients, with low-certainty evidence due to concerns regarding risk of bias and inconsistency. Mortality was similarly reduced, translating to 149 fewer deaths per 1,000 patients; however, the certainty of evidence was also rated as low, primarily due to very serious risk of bias. Seizure outcomes evidence was graded as very low due to serious risk of bias, inconsistency, and very serious imprecision. Lastly, ILE significantly reduced hospital length and was supported by moderate-certainty evidence. GRADE assessment of organophosphate, aluminum phosphide and antipsychotic independently maintained low and very low levels of certainty (Supplemental Fig. 19–21).

## Discussion

Our meta-analysis of 12 studies including 816 patients treated with ~ 280–500mL of primarily 20% SMOFlipid^®^ demonstrated improved clinical outcomes compared with standard resuscitative care in cases of acute poisoning from anti-psychotics, organophosphates, and aluminum phosphide. Treatment with ILE successfully reduced the odds of ICU admission and/or intubation for organophosphates and aluminum phosphide, contributing to the overall effect. Lipid emulsion also improved secondary outcomes, leading to a reduction in mortality for organophosphates and aluminum phosphide and a shortening of length of stay for organophosphates, aluminum phosphide and antipsychotics. Apart from length of stay, these outcomes were all rated with low or very low certainty based on GRADE assessments, driven by high or unclear levels of bias, lack of controls, low numbers of studies, and few total participants. These results are hypothesis-generating about the need for larger placebo-controlled trials, with a pre-specified dosing regimen and clinically meaningful outcome measures, including mortality, need for intubation and length of hospital stay.

The use of ILE in local anesthetic poisoning is well established with professional recommendations from multiple professional societies [33, 34]. However, the use of ILE in non-local anesthetic poisonings is more controversial, with representatives from multiple toxicological and poison control societies advocating against its use, except in extreme circumstances [16]. Since the lipid emulsion workgroup’s 2016 recommendations, the literature has evolved, with multiple randomized trials. A prior meta-analysis evaluated the impact of ILE on outcomes in organophosphate poisoning and demonstrated an improved overall prognosis [35]. We attempted to find papers from that review but could only verify two of the seven cited studies, raising concerns about the reliability of its conclusions. Two more recent publications analyzed ILE use in acute poisoning include a narrative review by Bilvanisi et al. [28] and a systematic review by Koh et al. [29]. The former similarly concluded that while ILE may be reasonable for adjunctive treatment of severe lipid-soluble drug toxicities, further clinical studies are necessary. The latter review found that patients who received ILE after sedative and antipsychotic overdose had greater improvements to their GCS over time. Koh et al. [29] attempted a meta-analysis but ultimately bypassed it due to non-uniform reporting of outcomes and marked heterogeneity in endpoints. The present analysis incorporates more recent reports, a wider range of toxic exposures, and the use of ICU admission as a primary outcome. Additionally, we identified consistent administration of lipid with 20% SMOFlipid^®^ as the primary formulation an initial dose of 100mL (1.5mL/kg) and total doses of 280–500 mL.

Overall, acute poisoning presents a global health challenge. In the United States alone, the National Poison Data System logged more than 2.4 million human exposures in 2024 with serious outcomes increasing by 4.08% per year since 2000 [36], highlighting the acuity of this issue. Internationally, the burden is even greater, with the 2021 Global Burden of Disease study reporting 3.58 million cases of non-carbon monoxide poisoning [37]. Intentional poisoning further contributes to these numbers, with one study approximating 110,000 deaths from pesticide self-poisonings each year from 2006 to 2015 [38]. Developing countries are disproportionately affected by poisonings, as there may be limited access to advanced medical care and ICU level care [39, 40]. As such, there is a need for treatment options to reduce catastrophic complications from poisoning.

A larger problem is the overall level of evidence and availability of antidotes for poisonings. The positive benefit of ILE in this meta-analysis derived primarily from studies of the treatment of organophosphate and aluminum phosphide overdoses, which are more specific to developing countries [41, 42]. For both agents, ILE demonstrated strong or very strong associations with inconsistencies in dosing, lack of controls, and variable risk of bias, reducing the certainty of recommendations. Atropine is the primary treatment for organophosphate toxicity, based on observational and mechanistic studies but without formal randomized controlled evidence [33, 43]. Professional societies and the World Health Organization also recommend oximes for organophosphate overdose [33] despite emerging signals for harm [44]. Conversely, aluminum phosphide lacks a specific antidote, contributing to a need for treatment that extends beyond “supportive care”. For aluminum phosphide poisoning, care primarily focuses on maintenance of blood pressure and prevention of acidosis. An inexpensive, widely available treatment with a specific dosing regimen would be very helpful in resource-constrained areas. Additionally, we observed a benefit to length of stay in the antipsychotic group, a more Western Problem [45, 46]. Like aluminum phosphide overdoses, antipsychotic poisoning is treated with supportive care, and clinicians would benefit from higher quality treatments. Lipid emulsion benefits from biological plausibility for scavenging [6], along with clinical data supporting both pharmacokinetic [7] and cardiovascular benefits [11]. This meta-analysis adds another layer to the literature supporting ILE, but only for specific indications, with the caveat that larger trials are needed, especially in Western Countries.

### Limitations

There are several limitations to our analysis. Each individual xenobiotic category included between two and four studies with 80 and 294 patients (816 patients total), limiting the robustness and generalizability of the pooled estimates. Second, approximately 30% of the studies included in the meta-analysis did not report the selected primary outcome. The pooling of studies caused “serious inconsistency” in the GRADE assessment, leading to very low certainty of evidence supporting reductions in ICU admission, mortality, and seizure reduction. The level of certainty was further confounded by the high or unclear risk of bias in most studies, warranting caution when interpreting results. The pooled estimates should be interpreted as an average effect across very different poisoning syndromes rather than evidence supporting lipid emulsion for xenobiotic poisoning in general. Next, our primary outcome was a composite of ICU admission and/or intubation which reflects institution specific decisions; but we coupled it with a secondary outcome of mortality which mirrored the benefit in the organophosphate and aluminum phosphide subgroups. Lastly, two studies, Basiouny et al. [22] and Elgazzar et al. [21], reported identical triglyceride values, which may reflect shared data sources and should be interpreted with caution. If these two studies shared data, inclusion of both would cause an overestimation of the pooled effects in our analysis. However, leave-one-out analysis of each of these studies did not change the results mitigating this concern. Finally, we did not investigate complications from ILE, but ILE therapy can interfere with lab samples [47] and large doses of ILE cause lipid emboli and other complications [48].

Several deviations from the preregistered protocol should be noted. We used the Cochrane RoB2 to provide a more comprehensive assessment of study quality. We also applied more appropriate meta-analytic methods including a random-effects model in the setting of high predicted heterogeneity. Heterogeneity and publication bias assessments were also revised: funnel plots, Egger’s regression, Galbraith plots, and L’Abbé plots were used in place of the initially proposed Doi plot and LFK index. Finally, a minimum of three studies were required for meta-analysis, a criterion not prespecified but adopted to ensure meaningful statistical interpretation. We did not analyze all outcomes due to insufficient data and a mismatch in measurement time points. Qualitative trends indicated that GCS improved faster with ILE treatment compared to standard care, potentially suggesting faster neurological recovery. Future trials should focus on consistent measurement of outcomes including serum triglycerides and blood pressure.

### Conclusion

In conclusion, this systematic review and meta-analysis demonstrated that 280–500 mL of ILE was associated with a decreased need for ICU admission and/or intubation and reduced mortality in organophosphate and aluminum phosphide toxicity, along with a decreased length of admission for antipsychotic, organophosphate and aluminum phosphide poisonings. However, the available evidence was limited by heterogeneity, low-quality studies, lack of placebo controls and divergent dosing schema. All these components contributed to low and very low certainty GRADE assessment, except for length of stay. The results of this study serve as hypothesis-generating information to guide future trials with pre-registered protocols, placebo controls, pre-specified dosing (280–500 mL 20% SMOFlipid^®^ or analogous), and clinically meaningful outcomes.

## Supplementary Information

Below is the link to the electronic supplementary material.

Supplementary Material 1 (DOCX 2.60 MB)

## References

[1] Gitman M, Fettiplace MR, Weinberg GL, Neal JM, Barrington MJ. Local Anesthetic Systemic Toxicity: A Narrative Literature Review and Clinical Update on Prevention, Diagnosis, and Management. Plast Reconstr Surg. 2019;144:783–95. . 10.1097/PRS.0000000000005989 31461049

[2] Fettiplace MR, McCabe DJ. Lipid emulsion improves survival in animal models of local anesthetic toxicity: a meta-analysis. Clin Toxicol. 2017;55:617–23. 10.1080/15563650.2017.1288911 28346007

[3] Boleyn J, Hardie K, Burt MR, Wieman S. Local anesthetic systemic toxicity during transversus abdominis plane block with liposomal bupivacaine. Am Surg. 2023;89:4252–4. 10.1177/00031348231206578. 37840256

[4] Lange DB, Schwartz D, DaRoza G, Gair R. Use of intravenous lipid emulsion to reverse central nervous system toxicity of an iatrogenic local anesthetic overdose in a patient on peritoneal dialysis. Ann Pharmacother. 2012;46. 10.1345/aph.1r298. 23191933

[5] Fettiplace MR, Weinberg G. The mechanisms underlying lipid resuscitation therapy. Reg Anesth Pain Med. 2018;43:138–49. 10.1097/AAP.0000000000000719. 29356774

[6] Fettiplace MR, Lis K, Ripper R, Kowal K, Pichurko A, Vitello D, et al. Multi-modal contributions to detoxification of acute pharmacotoxicity by a triglyceride micro-emulsion. J Control Release. 2015;198:62–70. 10.4135/9781412963947.n116 PMC4293282 25483426

[7] Dureau P, Charbit B, Nicolas N, Benhamou D, Mazoit JX. Effect of intralipid^®^ on the dose of ropivacaine or levobupivacaine tolerated by volunteers. Anesthesiology. 2016;125:474–83. 10.1097/ALN.0000000000001230 27404223

[8] Fettiplace MR, Ripper R, Lis K, Lin B, Lang J, Zider B, et al. Rapid cardiotonic effects of lipid emulsion infusion. Crit Care Med. 2013;41. 10.1097/CCM.0b013e318287f874. 23531591

[9] Enokiya T, Zhang E, Ikemura K, Muraki Y, Iwashita Y, Iwamoto T, et al. Effect of lipid emulsion infusion on paliperidone pharmacokinetics in the acute overdose rat model: A potential emergency treatment for paliperidone intoxication. Eur J Pharm Sci. 2017;109:217–22. 10.1016/j.ejps.2017.08.010 28821438

[10] Niiya T, Litonius E, Petäjä L, Neuvonen PJ, Rosenberg PH. Intravenous lipid emulsion sequesters amiodarone in plasma and eliminates its hypotensive action in pigs. Ann Emerg Med. 2010;56:402–e4082. 10.1016/j.annemergmed.2010.06.001 20868908

[11] Levine M, Brent J, Wiegand T, Maguire B, Cohen N, Vaerrier D, et al. Lipid emulsion therapy during management of the critically-ill poisoned patient: a prospective cohort study. Clin Toxicol. 2023;61:584–90. 10.1080/15563650.2023.2248372 37655788

[12] Fettiplace MR, Weinberg G, Chiang C, Nixon HC, Gitman M. Contemporary local anesthetic-associated adverse events and mortality: a pharmacovigilance analysis of a US reporting system. Br J Anaesth. 2025;135:1015–25. 10.1016/j.bja.2025.06.044. PMC12674073 40877109

[13] Fettiplace M, Weinberg G, Nixon H, Barabanova A, Chiang C, Gitman M. The impact of local anesthetic systemic toxicity advisories on reporting to the National Poison Data System (NPDS). Reg Anesth Pain Med. 2025. 10.1136/rapm-2025-106464 PMC13638343 40691088

[14] Fettiplace MR, Pichurko AB. Heterogeneity and bias in animal models of lipid emulsion therapy: a systematic review and meta-analysis. Clin Toxicol. 2021;59:1–11. 10.1080/15563650.2020.1814316. 33025830

[15] Cao D, Heard K, Foran M, Koyfman A. Intravenous Lipid Emulsion in the Emergency Department: A Systematic Review of Recent Literature. J Emerg Med. 2015;48:387–97. 10.1016/j.jemermed.2014.10.009 25534900

[16] Gosselin S, Hoegberg LC, Hoffman RS, Graudins A, Stork CM, Thomas SH, et al. Evidence-based recommendations on the use of intravenous lipid emulsion therapy in poisoning. Clin Toxicol. 2016;54:899–923. 10.1080/15563650.2016.1214275 27608281

[17] Chhabria BA, Bhalla A, Shafiq N, Kumar S, Dhibar DP, Sharma N. Lipid emulsion for acute organophosphate insecticide poisoning–a pilot observational safety study. Clin Toxicol. 2019;57:318–24. 10.1080/15563650.2018.1520997 30307350

[18] Pannu AK, Garg S, Bhalla A, Dhibar DP, Sharma N. Lipid emulsion for the treatment of acute organophosphate poisoning: an open-label randomized trial. Clin Toxicol. 2021. 10.1080/15563650.2021.2013496. 34928182

[19] Majeed S, Malik AH, Dar BA. Evaluation of intravenous lipid emulsion as a novel therapeutic antidote in severe organophosphorous poisoning- A prospective randomized, comparative study. Al Ameen J Med Sci. 2019;12:160–5.

[20] Guo J, Li Y, Sang Z. Analysis of therapeutic effect of lipid emulsion for liver function damage in acute severe organophosphorus pesticide poisoning. J Clin Emerg (China). 2015;16.

[21] Elgazzar FM, Elgohary MS, Basiouny SM, Lashin HI. Intravenous lipid emulsion as an adjuvant therapy of acute clozapine poisoning. Hum Exp Toxicol. 2021;40:1053–63. 10.1177/0960327120983873. 33401984

[22] Basiouny S, Elgohary M, Elgazzar F, Lashin H. Intravenous lipid emulsion as an adjuvant therapy of acute antipsychotic poisoning: a randomized controlled trial. Ain Shams J Forensic Med Clin Toxicol. 2022;38:19–32. 10.21608/ajfm.2022.212372.

[23] Kazemifar AM, Yazdi Z, Bedram A, Mahmoudi J, Ziaee M. Effects of Intravenous Lipid Emulsion on Tramadol-Induced Seizure; a Randomized Clinical Trial. Arch Acad Emerg Med. 2021;9:1–6. 10.22037/aaem.v9i1.1070. PMC8035696 33870207

[24] Behnoush AH, Alizadeh N, Emami M, Bazmi E, Alimohamadi Y, Behnoush B. Effects of Intravenous Lipid Emulsion Administration in Acute Tramadol Poisoning: A Randomized Controlled Trial. J Emerg Med. 2024;66:154–62. 10.1016/j.jemermed.2023.11.004 38309983

[25] Gheat HS, Fayed MM, Elgazzar FM, Draz EI, El-Kelany RS. The possible therapeutic role of intravenous lipid emulsion in acute aluminium phosphide poisoning: a randomized controlled clinical trial. Toxicol Res (Camb). 2024;13(3):tfae090. 10.1093/toxres/tfae090 PMC11170490 38883412

[26] ELabdeen S, Saad K, Oreby M, Elgazzar F. Assessment of Intravenous Lipid Emulsion as an Adjuvant Therapy in Acute Aluminum Phosphide Poisoning: A randomized Controlled Trial. Ain Shams J Forensic Med Clin Toxicol. 2020;34:51–68. 10.21608/ajfm.2020.68225.

[27] Singh Bhatia M, Attri R, Saroch A, Kumar Pannu A, Singla N, Chandrabhan Sharda S. Efficacy of intravenous lipid emulsion as an adjunctive therapy for acute aluminium phosphide poisoning: A randomized, open-label, pilot clinical trial. Eur Rev Med Pharmacol Sci. 2026;30:27–36. 10.26355/eurrev_202601_37646 41636291

[28] Bilvanisi S, Gülen M, Sabak M, Demiryürek Ş, Demiryürek AT. Intravenous Lipid Emulsion Therapy in Drug Overdose and Poisoning: An Updated Review. Eurasian J Med AVES. 2024;205–12. 10.5152/eurasianjmed.2024.24510. PMC11535339 39655869

[29] Koh J, Chalmers D, Hryniv R, King A, Cave G. Could Intravenous Lipid Emulsion Improve the Level of Consciousness in Acute Sedative and Antipsychotic Poisoning? A Review of Randomised Human Trials. Emerg Care Med MDPI AG. 2025;2:10. 10.3390/ecm2010010.

[30] Wan X, Wang W, Liu J, Tong T. Estimating the sample mean and standard deviation from the sample size, median, range and/or interquartile range. BMC Med Res Methodol. 2014. http://www.biomedcentral.com/1471-2288/14/135 10.1186/1471-2288-14-135 PMC4383202 25524443

[31] Minjun D. Clinical study of fat emulsion for acute severe organophosphorus poisoning. J Clin Emerg. 2013;14:546.

[32] Taftachi F, Sanaei-Zadeh H, Sepehrian B, Zamani N. Lipid emulsion improves Glasgow Coma Scale and decreases blood glucose level in the setting of acute non-local anesthetic drug poisoning - A randomized controlled trial. Eur Rev Med Pharmacol Sci. 2012;16:38–42. 22582483

[33] Cao D, Arens AM, Chow SL, Easter SR, Hoffman RS, Lagina AT, et al. Part 10: Adult and Pediatric Special Circumstances of Resuscitation: 2025 American Heart Association Guidelines for Cardiopulmonary Resuscitation and Emergency Cardiovascular Care. Circulation. 2025;152:S578–672. 10.1161/CIR.0000000000001380 41122889

[34] Neal JM, Barrington MJ, Fettiplace MR, Gitman M, Memtsoudis SG, Mörwald EE, et al. The Third American Society of Regional Anesthesia and Pain Medicine Practice Advisory on Local Anesthetic Systemic Toxicity. Reg Anesth Pain Med. 2018;43:113–23. 10.1097/AAP.0000000000000720. 29356773

[35] Yu S, Yu S, Zhang L, Gao Y, Walline J, Lu X, et al. Efficacy and outcomes of lipid resuscitation on organophosphate poisoning patients: A systematic review and meta-analysis. Am J Emerg Med. 2019;37:1611–7. 10.1016/j.ajem.2018.11.022 30527914

[36] Beuhler MC, Feldman R, Gummin DD, Mowry JB, Rivers LJ, Brown K, et al. 2024 Annual report of the National Poison Data System® (NPDS) from America’s Poison Centers®: 42nd annual report. Clin Toxicol. 2025;63:1029–280. 10.1080/15563650.2025.2571299 41432769

[37] Lei R, Yue C, Yue F, Gao H, He X, Yan Q, et al. Burden of non-CO poisoning in 204 countries and territories, 1990–2021: results from the global burden of disease studyBurden of non-CO poisoning in 204 countries and territories, 1990–2021: results from the global burden of disease study 2021. Front Public HealthFrontMediaSA. 2025;13:1620523. 10.3389/fpubh.2025.1620523 PMC12331598 40786150

[38] Mew EJ, Padmanathan P, Konradsen F, Eddleston M, Chang S, Sen, Phillips MR, et al. The global burden of fatal self-poisoning with pesticides 2006-15: Systematic review. J Affect Disord. 2017;93–104. 10.1016/j.jad.2017.05.002 28535450

[39] Murthy S, Leligdowicz A, Adhikari NKJ. Intensive care unit capacity in low-income countries: A systematic review. PLoS ONE. 2015;10. . 10.1371/journal.pone.0116949 PMC4305307 25617837

[40] Nawaz FA, Deo N, Surani S, Maynard W, Gibbs ML, Kashyap R. Critical care practices in the world: Results of the global intensive care unit need assessment survey 2020. World J Crit Care Med. 2022;11:169–77. 10.5492/wjccm.v11.i3.169 PMC9136725 36331973

[41] Eddleston M, Buckley NA, Eyer P, Dawson AH, Straub W. Management of acute organophosphorus pesticide poisoning. Lancet. 2008;371:597. 10.1016/S0140 PMC2493390 17706760

[42] Gurjar M, Baronia AK, Azim A, Sharma K. Managing aluminum phosphide poisonings. J Emerg Trauma Shock. 2011;4:378–84. 10.4103/0974-2700.83868. PMC3162709 21887030

[43] Lavonas EJ, Akpunonu PD, Arens AM, Babu KM, Cao D, Hoffman RS, et al. 2023 American Heart Association Focused Update on the Management of Patients With Cardiac Arrest or Life-Threatening Toxicity Due to Poisoning: An Update to the American Heart Association Guidelines for Cardiopulmonary Resuscitation and Emergency Cardiova. Circulation. 2023;148:e149–84. 10.1161/cir.0000000000001161 37721023

[44] Chauhan V, Goyal D, Thakur S, Galwankar S, Peredy TR. Therapeutic Interventions in Organophosphate Poisoning: An Umbrella Review of Systematic Reviews. Western J Emerg Med. 2026;27:819–30. 10.5811/westjem.50823 PMC13246183 42258859

[45] Levine M, Ruha A-M. Overdose of Atypical Antipsychotics Clinical Presentation, Mechanisms of Toxicity and Management. CNS Drugs. 2012;26:601–11. 10.2165/11631640-000000000-00000 22668123

[46] Handley SA, Every-Palmer S, Flanagan RJ. Antipsychotic-Related Fatal Poisoning, England and Wales, 1993–2019: The Impact of Second-Generation Antipsychotics. J Clin Psychopharmacol. 2021;41:650–7. 10.1097/JCP.0000000000001479 34668874

[47] Tan JG, Wong MS. Does the use of fish oil-based lipid emulsion in the clinical setting of total parenteral nutrition and lipid rescue therapy interfere with common laboratory analytes on Roche Cobas 6000Does the use of fish oil-based lipid emulsion in the clinical setting of total parenteral nutrition and lipid rescue therapy interfere with common laboratory analytes on Roche Cobas 6000? Ann Clin Biochem. 2021;58(3):220-229. 10.1177/0004563220986593 33353371

[48] Fettiplace MR, Akpa BS, Rubinstein I, Weinberg G. Confusion About Infusion: Rational Volume Guidelines for Intravenous Lipid Emulsion During Treatment of Oral Overdoses. Ann Emerg Med. 2015;66(2):185–8. 10.1016/j.annemergmed.2015.01.020 25737211

