# Supplementary material for: Systematic Review and Meta-Analysis of Lipid Emulsion for Non-Local Anesthetic Poisoning
Source: J Med Toxicol. 2026 Aug 27;22(4):461–71. doi: 10.1007/s13181-026-01151-7 (PMC13633206; doi:10.1007/s13181-026-01151-7)
Supplement: Supplementary file 1 [file 13181_2026_1151_MOESM1_ESM.docx]

**Systematic review and meta-analysis of lipid emulsion for xenobiotic poisoning**

Zhengxin Tang,^1*^ Megan Zhao^1*,^ Alexandra Barabanova^1^, Quincy McCrary^2^, Michael Fettiplace^1^

# **Supplemental Material**

# Search Strategy

**Filter: 01/01/2010-3/16/2026**

**Two searches were conducted: 10/21/2024 and 3/16/2026**

**PUBMED**

Search: ((((systemic toxicity) OR (toxicity)) OR (intoxication)) OR (poisoning)) AND ((Intralipid) OR (lipid emulsion) OR (smoflipid) OR (clinoleic) OR (nutralipid) OR (Omegaven)) AND (trial)

**EMBASE**

('systemic toxicity'/exp OR 'systemic toxicity' OR 'toxicity'/exp OR toxicity OR 'intoxication'/exp OR intoxication OR 'poisoning'/exp OR poisoning) AND ('intralipid'/exp OR intralipid OR 'lipid emulsion'/exp OR 'lipid emulsion' OR (('lipid'/exp OR lipid) AND ('emulsion'/exp OR emulsion)) OR 'smoflipid'/exp OR smoflipid OR 'clinoleic'/exp OR clinoleic OR 'nutralipid'/exp OR nutralipid OR 'omegaven'/exp OR omegaven) AND ('trial'/exp OR 'trial') AND [embase]/lim NOT ([embase]/lim AND [medline]/lim)

**WEB OF SCIENCE**

ALL=(systemic toxicity) OR ALL=( toxicity ) OR ALL=( intoxication ) OR ALL=(poisoning ) AND ALL=(Intralipid) OR ALL=(lipid emulsion) OR ALL=(smoflipid ) OR ALL=(clinoleic ) OR ALL=(nutralipid ) OR ALL (Omegaven)

AND ALL=(trials)

**SCOPUS**

( ( TITLE-ABS-KEY ( systemic AND toxicity ) OR TITLE-ABS-KEY ( toxicity ) OR TITLE-ABS-KEY ( intoxication ) OR TITLE-ABS-KEY ( poisoning ) ) ) AND ( ( TITLE-ABS-KEY ( lipid AND emulsion ) OR TITLE-ABS-KEY ( smoflipid ) OR TITLE-ABS-KEY ( clinoleic ) OR TITLE-ABS-KEY ( nutralipid ) OR TITLE-ABS-KEY ( omegaven ) ) )

<https://app.covidence.org/reviews/505333>

### **Supplemental Table 1: Additional Study Characteristics calculated in the first 72 hours following treatment. ***

| Publication | Timepoint (hours) | Control | | | | ILE | | | | |
| --- | --- | --- | --- | --- | --- | --- | --- | --- | --- | --- |
|  |  | GCS | SBP | MAP | DBP | GCS | SBP | MAP | DBP | Triglycerides |
| Behnoush 2024 | 0 | 9.5 | 127.7 |  | 83.2 | 10 | 129.7 |  | 80.5 |  |
|  | 12 | 10 | 129.9 |  | 82.4 | 12 | 120.8 |  | 77.1 |  |
|  | 24 | 12 | 131 |  | 85.4 | 14 | 123.4 |  | 78.9 |  |
| Pannu 2022 | 0 |  | 104.4 |  | 68.7 |  | 106.7 |  | 66.6 |  |
|  | 24 |  | 105.6 |  | 68.7 |  | 116.3 |  | 74.7 |  |
|  | 48 |  | 114.7 |  | 73.7 |  | 117 |  | 74 |  |
|  | 72 |  | 111.2 |  | 71.7 |  | 117.3 |  | 73.6 |  |
| Elgazzar 2021 | 0 | 8.5 |  |  |  | 8.8 |  |  |  | 87.2 |
|  | 6 | 9.2 |  |  |  | 13.1 |  |  |  |  |
|  | 12 | 9.6 |  |  |  | 14.3 |  |  |  | 100.2 |
| Gheat 2024 (moderate)* | 0 |  |  | 66.7 |  |  |  | 74.9 |  |  |
|  | 4 |  |  | 56.6 |  |  |  | 83.3 |  |  |
|  | 8 |  |  | 25.0 |  |  |  | 84.6 |  |  |
|  | 12 |  |  | 24.6 |  |  |  | 78.3 |  |  |
| Gheat 2024*(severe) | 0 |  |  | 23.3 |  |  |  | 41.7 |  |  |
|  | 4 |  |  | 48.3 |  |  |  | 70 |  |  |
|  | 8 |  |  | 16.6 |  |  |  | 76.6 |  |  |
|  | 12 |  |  | 16.6 |  |  |  | 60 |  |  |
| Taftachi 2012 | 0 | 110 |  |  |  |  | 101 |  |  |  |
|  | 6 | 117 |  |  |  |  | 112 |  |  |  |
| Basiouny2022** | 0 | 9 |  |  |  | 10.1 |  |  |  | 87.2 |
|  | 6 | 10 |  |  |  | 15 |  |  |  | 100.2 |
|  | 12 | 14 |  |  |  | 15 |  |  |  |  |

***No additional data for Kazemifar 2021, Majeed 2019, Elabdeen 2020, Guo 2015, Minjun 2013 or Bhatia 2026**

ILE: Intravenous lipid emulsion; GCS: Glasgow coma-score; SBP: systolic blood pressure, MAP: Mean arterial pressure; DBP: Diatolic blood pressure

*MAP reported as median

**Basiouny 2022 GCS score reported as median, except for GCS(0), which is reported as mean.

**Supplemental Table 2: Additional treatments and placebo.**

| **Publication** | **Placebo or Control treatment** | **Other treatments (for both groups)** | **Blinding** |
| --- | --- | --- | --- |
| ***Tramadol*** | | | |
| *Behnoush 2024* | 0.9% saline | Naloxone, IV fluids, benzodiazepines. IV sodium bicarbonate | Double-blind (participants & providers) |
| *Kazemifar 2021* | - | Gastric decontamination, adequate supportive care | Single-blind (participants) |
| ***Organophosphate*** | | | |
| *Pannu 2022* | 0.9% saline | Atropine, gastric decontamination | Unblinded |
| *Majeed 2019* | Pralidoxime | Atropine, gastric decontamination | Unblinded |
| *Guo 2015* | - | Atropine, gastric decontamination, diuresis, hemoperfusion, pralidoxime, penehyclidine, glutathione | Not noted |
| *Minjun 2013* | - | Atropine, gastric lavage, pralidoxime, hemoperfusion, | Not noted |
| ***Antipsychotics*** | | | |
| *Elgazzar 2021* | - | Gastric decontamination, IV fluids | Single-blinded (presumed as participants) |
| *Basiouny 2022* | - | Magnesium sulfate, benzodiazepines, gastric decontamination | Single-blinded (presumed as participants) |
| ***Aluminium Phosphide*** | | | |
| *Gheat 2024* | - | IV fluids, norepinephrine, gastric decontamination, IV sodium bicarbonate | Single-blinded (participants) |
| *Elabdeen 2020* | - | IV fluids, norepinephrine, gastric decontamination, IV sodium bicarbonate, Magnesium sulfate | Not noted |
| *Bhatia 2026* | 0.9% saline | IV fluids, vasopressors, inotropes, gastric decontamination, magnesium sulfate, IV sodium bicarbonate, proton pump inhibitor | Not noted |
| **Mixed** | | | |
| *Taftachi 2012* | - | IV fluids, vasopressors, inotropes, IV sodium bicarbonate | Not noted |

**Supplemental Figure 1:** Risk of bias 2 (ROB2) Assessment

### **Supplemental Figure 2: Funnel plot for ICU admission**

### **Supplemental Figure 3: Galbraith plot for ICU admission**

### **Supplemental Figure 4: L'Abbé Plot for ICU admission**

**Supplemental Figure 5**

**Supplemental Figure 6**

θ

### **Supplemental Figure 7: Funnel plot for mortality**

### **Supplemental Figure 8: Galbraith plot for mortality**

### **Supplemental Figure 9: L’Abbé plot for mortality**

### **Supplemental Figure 10**

### **Supplemental Figure 11: Funnel plot of length of admission**

### **Supplemental Figure 12: Galbraith plot of length of admission**

### **Supplemental Figure 13: Forest plot of length of stay (Interpolated data according to Wan et al removed)**

### **Supplemental Figure 14: Forest plot of seizures**

### **Supplemental Figure 15: Funnel plot for seizures**

### **Supplemental Figure 16: Galbraith plot for seizures**

### **Supplemental Figure 17: L’Abbé plot for seizures**

### **Supplemental Figure 18: GRADE Assessment:** ILE compared to standard care for non-local anesthetic poisoning

| **Certainty assessment** | | | | | | | **№ of patients** | | **Effect** | | **Certainty** | **Importance** |
| --- | --- | --- | --- | --- | --- | --- | --- | --- | --- | --- | --- | --- |
| **№ of studies** | **Study design** | **Risk of bias** | **Inconsistency** | **Indirectness** | **Imprecision** | **Other considerations** | **ILE** | **standard care** | **Relative (95% CI)** | **Absolute (95% CI)** |  |  |
| **ICU admission** | | | | | | | | | | | | |
| 8 | randomised trials | serious^a^ | serious^b^ | serious^c^ | serious^d^ | none | 79/185 (42.7%) | 112/184 (60.9%) | **RR 0.70** (0.53 to 0.93) | **183 fewer per 1,000** (from 286 fewer to 43 fewer) | ⨁◯◯◯ Very low^a,b,c,d^ |  |
| **Mortality** | | | | | | | | | | | | |
| 8 | randomised trials | very serious^e^ | not serious | serious^c^ | not serious | none | 63/251 (25.1%) | 113/265 (42.6%) | **RR 0.65** (0.50 to 0.78) | **149 fewer per 1,000** (from 213 fewer to 94 fewer) | ⨁◯◯◯ Very low^c,e^ |  |
| **Seizures** | | | | | | | | | | | | |
| 3 | randomised trials | serious^a^ | serious^f^ | serious^c^ | very serious^d^ | strong association dose response gradient | 16/123 (13.0%) | 46/122 (37.7%) | **RR 0.27** (0.06 to 1.26) | **275 fewer per 1,000** (from 354 fewer to 98 more) | ⨁◯◯◯ Very low^a,c,d,f^ |  |
| **Length of admission** | | | | | | | | | | | | |
| 6 | randomised trials | serious^a^ | not serious | serious^c^ | serious^d^ | very strong association^g^ | 174 | 172 | - | MD **34.2 hours higher** (26 higher to 42 higher) | ⨁⨁⨁◯ Moderate^a,c,d,g^ |  |

**CI:** confidence interval; **MD:** mean difference; **RR:** risk ratio

#### Explanations

**a.** Most studies with "some concerns" and a few "high risk". ; **b.** I2 =32% of between group differences significant (p=0.02)

**c.** Lack of placebos, different dosing regimens and different toxicities; **d.** Few studies, with low total number of patients

**e.** Most studies with high risk in RoB2; **f.** tau^2 = 0.94 with the possibility that true effects vary substantially accross studies

**g.** Concern that two studies were imported the data from the same patients.

**Supplemental Figure 19: GRADE Assessment;** ILE compared to standard of care for organophosphate poisoning

| **Certainty assessment** | | | | | | | **№ of patients** | | **Effect** | | **Certainty** | **Importance** |
| --- | --- | --- | --- | --- | --- | --- | --- | --- | --- | --- | --- | --- |
| **№ of studies** | **Study design** | **Risk of bias** | **Inconsistency** | **Indirectness** | **Imprecision** | **Other considerations** | **ILE** | **standard of care** | **Relative (95% CI)** | **Absolute (95% CI)** |  |  |
| **ICU Admission** | | | | | | | | | | | | |
| 2 | randomised trials | very serious | not serious | serious^a^ | serious^b^ | none | 53/73 (72.6%) | 60/72 (83.3%) | **RR 0.85** (0.75 to 0.96) | **125 fewer per 1,000** (from 208 fewer to 33 fewer) | ⨁◯◯◯ Very low^a,b^ |  |
| **Mortality** | | | | | | | | | | | | |
| 4 | randomised trials | very serious^c^ | serious^d^ | serious^a^ | serious^b^ | strong association | 18/146 (12.3%) | 36/148 (24.3%) | **RR 0.50** (0.30 to 0.83) | **122 fewer per 1,000** (from 170 fewer to 41 fewer) | ⨁◯◯◯ Very low^a,b,c,d^ |  |
| **Length of stay** | | | | | | | | | | | | |
| 3 | randomised trials | very serious^c^ | not serious | serious^a^ | serious^b^ | very strong association | 123 | 126 | - | MD **28.6 hours more** (14 more to 43 more) | ⨁⨁◯◯ Low^a,b,c^ |  |

**CI:** confidence interval; **MD:** mean difference; **RR:** risk ratio

#### Explanations

a. Different controls; pralidoxime and volume

b. Low number of studies and total participants

c. High RoB2 judgement for multiple studies

d. I2=0 but some trials without significant results

**Supplemental Figure 20: GRADE Assessment** ILE compared to standard care for aluminum phosphide poisoning

| **Certainty assessment** | | | | | | | **№ of patients** | | **Effect** | | **Certainty** | **Importance** |
| --- | --- | --- | --- | --- | --- | --- | --- | --- | --- | --- | --- | --- |
| **№ of studies** | **Study design** | **Risk of bias** | **Inconsistency** | **Indirectness** | **Imprecision** | **Other considerations** | **ILE** | **standard care** | **Relative (95% CI)** | **Absolute (95% CI)** |  |  |
| **ICU Admission** | | | | | | | | | | | | |
| 3 | randomised trials | serious | not serious | serious^a^ | serious^b^ | strong association | 17/57 (29.8%) | 36/57 (63.2%) | **RR 0.46** (0.30 to 0.70) | **341 fewer per 1,000** (from 442 fewer to 189 fewer) | ⨁⨁◯◯ Low^a,b^ |  |
| **Mortality** | | | | | | | | | | | | |
| 4 | randomised trials | serious^c^ | not serious | serious^a^ | serious^b^ | none | 45/105 (42.9%) | 77/107 (72.0%) | **RR 0.65** (0.47 to 0.88) | **252 fewer per 1,000** (from 381 fewer to 86 fewer) | ⨁◯◯◯ Very low^a,b,c^ |  |

**CI:** confidence interval; **RR:** risk ratio

#### Explanations

a. Lack of placebo controls for all studies. Different dosing schema for all studies.

b. Low number of studies and total participants

c. RoB2 demonstrates some concerns for all studies

**Supplemental Figure 21: GRADE Assessment”** ILE compared to standard of care for antipsychotic poisoning

| **Certainty assessment** | | | | | | | **№ of patients** | | **Effect** | | **Certainty** | **Importance** |
| --- | --- | --- | --- | --- | --- | --- | --- | --- | --- | --- | --- | --- |
| **№ of studies** | **Study design** | **Risk of bias** | **Inconsistency** | **Indirectness** | **Imprecision** | **Other considerations** | **ILE** | **standard of care** | **Relative (95% CI)** | **Absolute (95% CI)** |  |  |
| **Length of stay** | | | | | | | | | | | | |
| 2 | randomised trials | serious^a^ | not serious | serious^b^ | serious^c^ | publication bias strongly suspected very strong association^d^ | 40 | 40 | - | MD **33.6 hours more** (18 more to 49 more) | ⨁⨁◯◯ Low^a,b,c,d^ |  |

**CI:** confidence interval; **MD:** mean difference

#### Explanations

a. Some concerns noted for all studies

b. Lack of placebo

c. small number of studies with low number of patients

d. Concern for shared patients between both trials
